# Oxidative Stress and Genotoxicity of Long-Term Occupational Exposure to Low Levels of BTEX in Gas Station Workers

**DOI:** 10.3390/ijerph13121212

**Published:** 2016-12-06

**Authors:** Feng Xiong, Qin Li, Bo Zhou, Jiongli Huang, Guiqiang Liang, Li’e Zhang, Shuyan Ma, Li Qing, Linhan Liang, Jing Su, Xiaowu Peng, Qin Li, Yunfeng Zou

**Affiliations:** 1 Department of Toxicology, School of Public Health, Guangxi Medical University, Nanning 530021, China; xiongmimi91@163.com (F.X.); liqin_1229@163.com (Q.L.); lgq26855@163.com (G.L.); 15296543598@163.com (S.M.); 2 Research Center for Regenerative Medicine, Guangxi Medical University, Nanning 530021, China; gxzhoubo520@126.com; 3 Atmospheric Environment Research Center, Scientific Research Academy of Guangxi Environmental Protection, Nanning 530021, China; huangjiongli123@163.com; 4 Department of Occupational and Environmental Health, School of Public Health, Guangxi Medical University, Nanning 530021, China; zhang-121704151010@163.com (L.Z.); llh_cool@126.com (L.L.); 5 Department of Epidemiology and Health Statistics, School of Public Health, Guangxi Medical University, Nanning 530021, China; ql871126@163.com (L.Q.); smiles-sj@163.com (J.S.); 6 Center for Environmental Health Research, South China Institute of Environmental Sciences, Ministry of Environmental Protection, Guangzhou 510655, China; pengxiaowu@scies.org; 7 Department of Ophthalmology, First Affiliated Hospital of Guangxi Medical University, Nanning 530021, China

**Keywords:** antioxidant, BTEX, comet assay, DNA damage, micronucleus test, refueling workers

## Abstract

Atmospheric benzene, toluene, ethylbenzene, and xylenes (BTEX) can lead to multiple health injuries. However, what remains uncertain is the effect of long-term exposure to low levels of BTEX. Thus, we determined the BTEX levels in the air from the refueling and office areas in gas stations. Then we collected workers’ (200 refueling vs. 52 office workers) peripheral blood samples to analyze the serum total-superoxide dismutase (T-SOD), glutathione (GSH), malondialdehyde (MDA), and 8-hydroxydeoxyguanosine (8-OHdG) levels. DNA damage was analyzed by the comet assay and micronucleus test in buccal epithelial cells. We found that the levels of BTEX in refueling areas were significantly higher than those in office areas (*p* < 0.001). The serum T-SOD and GSH of refueling workers were significantly lower than those in office workers (*p* < 0.001). By contrast, the serum MDA and 8-OHdG of refueling workers were significantly higher than those of office workers (*p* < 0.001, MDA; *p* = 0.025, 8-OHdG). Furthermore, tail and Olive tail moments in refueling workers were longer (*p* = 0.004, tail moment; *p* = 0.001, Olive tail moment), and the micronucleus rate was higher (*p* < 0.001) than those in office workers. Taken together, long-term exposure to low levels of BTEX may reduce the antioxidant ability and increase the risk of DNA damage in refueling workers of gas stations.

## 1. Introduction

With the rapid development of the global economy and industrialization, many developing countries, including China, have experienced decades of rapid growth in the consumption of petroleum products for a certain number of automobiles. In addition, vehicle emission exhaust has become a growing source of volatile organic carbon (VOC) emissions [1,2]. Among the VOC pollutants, particular research and environmental attention has been given to benzene, toluene, ethylbenzene, and three xylene isomers (o-xylene, m-xylene, and p-xylene) (BTEX). BTEX can be extracted from coal tar and petroleum, and used as a raw material and solvent in the chemical industry. Moreover, BTEX are ubiquitous environmental contaminants and components of cigarette smoke [3]. However, a major source of BTEX found in cities is gasoline evaporation and vehicle emissions. This is because exhaust from fuel oil combustion contributes the most to BTEX concentrations in the atmospheric environment [4].

Managerial supervision has been applied to reduce the environmental and occupational exposures of BTEX. Taking benzene as an example, the Occupational Safety and Health Administration (OSHA) approved 32.5 mg/m^3^ (10 ppm) as the permissible exposure limit of benzene exposure in 1971, but this was later modified to 3.25 mg/m^3^ (1 ppm) in 1987 [5,6]. This mandatory limit is being used to this day. Nevertheless, several lines of evidence suggest that BTEX can still be found mainly at low concentrations below the standard levels in many workplaces, whether in developed countries or in developing countries [7,8,9,10,11]. Hence, the influence of long-term exposure to low levels of BTEX on human health effects has become an area of considerable research interest. However, a related population study in fuel-filling station attendants (FFSAs), particularly in developing countries, remains largely unexplored.

FFSAs can be exposed to BTEX from fuel and vehicular exhausts that enter their respiratory system [12]. BTEX can affect human health and cause multi-organ damage, including the central nervous, hemopoietic, and reproductive systems. Benzene has been classified as a human carcinogen by the United States Environmental Protection Agency (USEPA) [13]. Exposure to high levels of benzene for a short time can lead to toxicity or death, whereas prolonged exposure to low levels can result in bone marrow abnormality, leukemia, and aplastic anemia [14]. Workers exposed to benzene concentrations of 30 ppm and 650 ppm for three months to 17 years showed increased incidences of pancytopenia [15,16,17]. Meanwhile, the USEPA stated that the carcinogenic potential of toluene cannot be evaluated because of insufficient information [18]. Chronic inhalation of low levels of toluene, ethylbenzene, and xylene can have harmful effects on the central nervous system (e.g., neurasthenic syndrome). However, long-term exposure of workers to low levels of benzene but no other compounds of the BTEX group has a positive correlation with hematologic diseases, including aplastic anemia and leukemia.

Despite the harmful effects of BTEX, the roles of its mechanism of action and the concentration-response relationship in cases of low-level and long-term BTEX exposure to humans have yet to be addressed. BTEX can be oxidized in the body to form less toxic or non-toxic derivates. During this biochemical process, glutathione (GSH) and superoxide dismutase (SOD) protect cells from superoxide toxicity. Moreover, malondialdehyde (MDA) is formed, which can measure the level of oxidative stress in an organism. It also reacts with deoxyadenosine and deoxyguanosine in DNA, thus forming DNA adducts which are mutagenic.

Furthermore, BTEX metabolites can be oxidized to highly reactive intermediates, which interact with cellular macromolecules that can be detected by comet assay, including DNA strand breaks. The oxidative damage by free radicals in DNA molecules can also be reflected in the serum level of 8-hydroxydeoxyguanosine (8-OHdG). Therefore, the levels of 8-OHdG in serum and comet assays have been used to reveal DNA damage. The genotoxic damage of chemicals in humans can also be examined via a micronucleus test (MNT) in peripheral blood lymphocytes, which has been a reliable method for genotoxic carcinogens. MNT in buccal epithelial cells (BECs) is a much more convenient and economical method. However, few studies have examined whether it could be used as a reliable assay to determine genetic damage in humans, as in the case of gas station workers exposed to low levels of BTEX.

Therefore, our study aimed to (1) detect the concentration of BTEX in the air of gas stations in Nanning, China; and (2) analyze the oxidative stress and genotoxicity of the refueling workers who have had long-term and low-level exposure to BTEX in gas stations by determining the serum total-superoxide dismutase (T-SOD), MDA, GSH, and 8-OHdG contents using MNT in BECs and the comet assay in lymphocytes, respectively; and (3) evaluate whether MNT in BECs could be a reliable method for testing the DNA damage in BTEX-exposed workers.

## 2. Materials and Methods

### 2.1. Study Population

The study population consisted of 252 gas station workers working and living in Nanning City. Among these workers, 200 are refueling workers who are mainly refueling motor vehicles and are continually in contact with gasoline, and the remaining 52 are office workers who work and spend their time indoors. The basic and occupational data on these workers were acquired through personal interviews. Age, gender, occupation, exposure time, smoking, and drinking behavior were included in our questionnaire. The above characteristics of all subjects are summarized in Table 1.

### 2.2. Study Sites and Sampling

Nanning, the capital of Guangxi Province and a megacity located in the southwest part of China, is a regional international city of the China-Association of South East Asian Nations. In this city, motor vehicle population has increased rapidly, with numbers reaching as much as 1.756 million in 2015.

Air samples were collected in November 2012. Thirteen gas stations were randomly selected from six administrative zones of Nanning as occupational air sampling points. The samplers in each gasoline refueling areas were placed near to and at the downwind area of the fuel pumps according to the wind direction. The samplers in each office areas were placed in the middle of the offices. All the samplers were placed 1.5 m above the ground. At each sampling point, air samples were collected in 3.2 L pre-cleaned SUMMA canisters with flow regulators. Sampling was performed at 8:30–9:30 and 17:30–18:30, which are the rush periods at all sites for one working day.

Three milliliters of peripheral vein blood samples were collected from each of the 252 workers in gas stations during the same period. Normal heparin was used as anticoagulant. Serum was separated via centrifugation and stored in the refrigerator at −80 °C.

### 2.3. Sample Analysis

The occupational air samples collected from the gas stations were pre-concentrated in a concentrator (Entech7100A instrument, Simi Valley, CA, USA). 300 mL of air samples were extracted from each concentrator and determined by using an Agilent 7890A-5975C chromatography/mass spectrometry instrument (Agilent Technologies, Inc., Palo Alto, CA, USA). The initial temperature was set at 35 °C for 3 min, increased to 120 °C at a rate of 5 °C/min, further raised to 250 °C at a rate of 10 °C/min, and remained unchanged for 20 min. The relative standard deviation for repeatability in air samples was ≤15%. The detection limit of the procedure for benzene, toluene, Ethyl-benzene, p/m-xylene, and o-xylene were 0.03 μg/m^3^, 0.01 μg/m^3^, 0.02 μg/m^3^, 0.18 μg/m^3^, and 0.02 μg/m^3^, respectively.

### 2.4. Oxidative Stress Indicators Determination

T-SOD assay kits (Hydroxylamine method) were used to measure the activities of serum T-SOD, (Nanjing Jiancheng, Nanjing, China) and values were expressed as U/mL. GSH assay kits (Spectrophotometric method) were used to measure the levels of serum GHS (Nanjing Jiancheng, Nanjing, China) and values were expressed as mg/L. MDA assay kits (TBA method) were used to measure the levels of serum MDA (Nanjing Jiancheng, Nanjing, China) and values were expressed as μmol/L. Next, 8-OHdG Enzyme Linked Immunosorbent Assay kits (eBioscience, San Diego, CA, USA) were used to measure the levels of serum 8-OHdG and the values were expressed as ng/mL.

### 2.5. Comet Assay

A total of 50 lymphocytes were separated and collected from whole blood samples, according to the method of Singh [19] with slight modifications. Each sample was examined on 50 cells and randomly photographed under a fluorescent microscope (Nikon Eclipse E200, Tokyo, Japan). The images were measured using IMI1.0 single-cell gel electrophoresis analysis software. DNA damage was quantified by using the tail moment (TM) and Olive tail moment (OTM).

### 2.6. MNT in BECs

MNT was carried out on 200 refueling workers and 52 office workers. We followed the method described by Stich [20] with slight modifications [21]. Before sampling, all workers were required to rinse their mouth with clean water three or four times. Surface cells were scraped from their buccal mucosa with sterile spatula. A total of 1000 BECs were screened per subject, after which the micronucleus rate per 1000 cells was calculated. Scoring of micronuclei was performed manually using Olympus BX 51 fluorescent microscope.

### 2.7. Statistical Analysis

Double entry and validation were performed through the Epidata 3.0, and statistical analysis was performed by using the statistical package SPSS16.0. For descriptive analysis, results were presented as median (range) or mean ± SD. For further analysis, Wilcoxon signed-rank test was used to compare BTEX concentrations of refueling and office areas air samples. Wilcoxon rank test was used to calculate the variance of GSH, MDA, and 8-OHdG levels in serum, as well as the activities of T-SOD, TM, OTM, and micronucleus rate (BECs) in both the refueling and office workers. Multiple linear regression was used to evaluate the effect of group variables and personal characteristics (age, gender, years of exposure, and smoking and drinking habits) on T-SOD, GSH, MDA, 8-OHdG, micronucleus rate, TM and OTM as dependent variables. A *p*-value of <0.05 (two-tailed) was considered statistically significant.

### 2.8. Ethical Statements

All subjects gave their informed consent for inclusion before they participated in the study. The study was conducted in accordance with the Declaration of Helsinki, and the protocol was approved by the medical ethics committee of Ethics and human subjects committee of Guangxi Medical University, and the number/ID of the approval is (2014)LUNSHEN[KE]DI(125)HAO.

## 3. Results

### 3.1. Demographic and Occupational Characteristics, and Smoking and Drinking Habits between Refueling and Office Workers in the Gas Stations

The demographic and occupational characteristics of the study population are reported in Table 1. A total of 200 refueling and 52 office workers were investigated in 13 gas stations in Nanning. Among refueling workers, 98 (49%) were males and 102 (51%) were females; the average age was 33.1, and the average years of exposure were 8.7. Among office workers, 23 (44.2%) were males and 29 (55.8%) were females; the average age was 34.3, and the average years of exposure were 10.6. No significant difference in age (*t* = 1.1, *p* = 0.272) and gender (χ^2^ = 0.376, *p* = 0.540) was found between refueling and office workers. However, a significant difference was observed in years of exposure between refueling and office workers (*t* = 2.0, *p* = 0.047). No significant differences in terms of smoking (χ^2^ = 0.103, *p* = 0.748) or drinking (χ^2^ = 0.158, *p* = 0.691) habits were found between refueling and office workers.

### 3.2. BTEX Levels of Refueling and Office Area Air Samples

The results of BTEX levels in refueling and office area air samples are reported in Table 2. In the refueling area, the average levels of benzene, toluene, ethylbenzene, m-xylene, p-xylene, and o-xylene were 60.03, 317.76, 113.85, 40.91, 57.51, and 37.18 μg/m^3^, respectively. In the office area, the average levels of benzene, toluene, ethylbenzene, m-xylene, p-xylene, and o-xylene were 16.86, 89.93, 12.09, 28.44, 10.62, and 15.67 μg/m^3^, respectively. In both refueling and office areas of the sampled gas stations, the average BTEX concentrations in the air were ranked in the following order: toluene > m-xylene > benzene > o-xylene > ethylbenzene > p-xylene. Statistical analysis of the data showed that the concentrations of BTEX compounds of occupational air samples in the refueling areas were all significantly (*p* < 0.001) higher than those of the air samples collected from the office area.

### 3.3. Oxidative Stress Indicators in Refueling and Office Workers

The activities of T-SOD and the GSH levels were significantly ($z_{T-SOD}$ = −6.303, $z_{GSH}$ = −3.885, *p* < 0.001) decreased in refueling workers compared with office workers, while the MDA and 8-OHdG levels were significantly elevated ($z_{MDA}$ = −5.864, $z_{8–OHdG}$ = −6.047, *p* < 0.001), as reported in Table 3.

### 3.4. MNT and Comet Assay in Refueling and Office Workers

The micronucleus rate in refueling workers was significantly higher than that of office workers ($z_{\mathrm{Micronucleus}\;\mathrm{rate}}$ = −4.123, *p* < 0.001). Similarly, TM and OTM were significantly longer in refueling workers compared with those in the office workers ($z_{TM}$ = −2.779, *p* = 0.005; $z_{OTM\;}$ = −3.496, *p* < 0.001), as reported in Table 3.

### 3.5. Multiple Linear Regression for T-SOD, GSH, MDA, 8-OHdG, Micronucleus Rate, TM, and OTM in Refueling and Office Workers

The results of the multiple linear regression analysis are reported in Table 4. For T-SOD, GSH, MDA, 8-OHdG, the micronucleus rate, TM, and OTM, both models were significant, with *R^2^* values of 0.152, 0.094, 0.087, 0.091, 0.214, 0.035, and 0.044, respectively. After controlling for the confounding factors (age, gender, years of exposure, and smoking and drinking habits), T-SOD, GSH, MDA, 8-OHdG, TM, and OTM were influenced by the groups, the micronucleus rate was influenced by the groups and years of exposure. The activities of T-SOD and levels of GSH in refueling workers were significantly higher than in office workers. On the contrary, the levels of MDA, 8-OHdG, the micronucleus rate, TM, and OTM in refueling workers were significantly lower than in office workers. In addition, the micronucleus rate was positively correlated with years of exposure.

## 4. Discussion

BTEX comprise the most important components of gasoline and can be found at low levels in gasoline stations such as those reported earlier in other studies [10,22,23]. For benzene, toluene, ethylbenzene, and xylene, the exposure limits of the time weighted average, which are recommended by the National Institute for Occupational Safety and Health, are 0.1 (325 μg/m^3^), 100 (375 mg/m^3^), 100 (435 mg/m^3^), and 100 ppm (435 mg/m^3^ for three isomers), respectively [24,25,26,27]. In the present study, the mean BTEX concentrations in the air were 60.03, 317.76, 113.85, 40.91, 57.51, and 37.18 μg/m^3^, respectively, which are much lower than the current occupational limit values. However, the daily mean concentrations of BTEX compounds in the ambient air of the refueling areas were significantly higher compared with those in the office areas. This finding indicated that refueling workers were exposed to higher levels of BTEX compared with office workers via inhalation because BTEX concentrations significantly vary between the refueling and office areas. Similarly, air monitoring for benzene in petrol pumps from northern India also revealed significantly higher benzene levels [28]. For all practical reasons, fixed environmental monitoring was performed to characterize the exposure to BTEX in our study, and the levels of BTEX that FFSAs may actually be exposed to could be underestimated. According to previous studies [29] in which both ambient and personal breathing zone air sampling were conducted, a higher level of BTEX was found in the personal breathing zone air when compared to the ambient air. However, the variance of BTEX levels between the ambient and personal breathing zone air was small.

Furthermore, the concentrations of benzene, toluene, and m-xylene made up the greatest percentage of BTEX in the air of the gas station in this study. Toluene can be used as an octane booster in gasoline fuels, thus it is often used as a substitute for benzene. Nevertheless, toluene is less toxic than benzene. Mixed xylenes are used to blend into the gasoline, and 40%–65% m-xylene is typically present in mixed xylenes. That may explain why the m-xylene is in a majority of BTEX in the air samples among the three isomers of the xylene. However, mixed xylenes are classified as a Group D substance by USEPA which means they are not classifiable as to human carcinogenicity [30].

As a consequence, the reduction of the antioxidant ability and the genetic damage induced by low levels of BTEX were found in the refueling workers of gas stations in the present study. The T-SOD activities and GSH levels were significantly lower in refueling workers than in office workers, whereas the MDA and 8-OHdG levels showed the opposite trends. Our results are also supported by investigations on oxidative stress detected in various occupational exposures [31,32,33]. Comet parameters were used to study DNA damage, and significant increases in TM and OTM were noted in refueling workers. This result suggested that exposure to BTEX in air may increase the DNA damage in refueling workers, and the comet assay could be a useful detection method for occupational exposure to BTEX via inhalation. Besides, there are animal experiments suggesting that long-term VOC inhalation, including BTEX inhalation at low levels, can cause oxidative stress and a genotoxicity response in mice [34]. Results showed that exposure to BTEX (indoor air quality standard dose) results in oxidative lung damage, which is supported by significant changes in GSH and MDA. BTEX exposure also induces significant DNA damage in the liver. Because of a large sample size, immunoenzymatic assays were used to determine the biomarkers of the oxidative stress and genotoxicity of gas station workers in our study, the results of which may be less sensitive and specific since they could be affected by some exogenous factors, especially when compared to gas chromatography/mass spectrometry or liquid chromatography-mass spectrometry methods. However, immunoenzymatic assays can still be used to test the biomarkers when they can meet the requirements of accuracy and precision, which means more attention should be paid to the accuracy of methods in our future research.

Meanwhile, a significant increase of the micronucleus frequency in BECs in refueling workers was also found. Notably, we observed a consistent trend with the micronucleus frequencies in previous studies, which were tested in peripheral blood lymphocytes [28,35]. Therefore, our study may suggest that the micronucleus ratio of BECs could also reflect the damage of the genetic material. The MNT in BECs could serve as a more economical and convenient assay in detecting genotoxic damage caused by exposure to BTEX.

## 5. Conclusions

In conclusion, our results showed that despite the fact that the BTEX levels are below the current occupational exposure limits, the decrease of antioxidant enzymes and the increase of genotoxic damage could still be observed in refueling workers. Therefore, our results suggested that the end points of oxidative stress and genotoxicity are both useful biomarkers (of exposure and effect, respectively) of occupational exposure to low levels of BTEX. The results also suggested that MNT in BEC could also be a reliable assay in detecting the DNA damage in BTEX-exposed workers of gas stations. However, for the present results, we still need large, retrospective cohort studies with sufficient follow-up to understand the effects of long-term exposure to low levels of BTEX, specifically in developing countries to ensure occupational health.

## Figures and Tables

**Table 1:** Demographic characteristics of refueling workers and office workers.

Confounding Factor	Refueling Workers (*n* = 200)		Office Workers (*n* = 52)		χ^2^ (*t*)	*p*
Age (years) (Mean ± SD)	33.1	7.1	34.3	6.2	1.1	0.272
Years of exposure (Mean ± SD)	8.7	6.0	10.6	6.5	2.0	0.047
Gender, *n* (%)						
Male	98	49.0	23	44.2	0.376	0.540
Female	102	51.0	29	55.8		
Smoking, *n* (%)						
Yes	57	28.5	16	30.8	0.103	0.748
No	143	71.5	36	69.2		
Alcohol consumption, *n* (%)						
Yes	83	41.5	20	38.5	0.158	0.691
No	117	58.5	32	61.5		

SD: Standard Deviation.

**Table 2:** BTEX levels in the breathing zone air of the office area and refueling area (μg/m^3^, *n* = 26).

Compound	Refueling Area				Office Area				*z*	*p*
	Mean	Median	*P*_25_	*P*_75_	Mean	Median	*P*_25_	*P*_75_		
Benzene	60.03	41.89	26.54	60.36	16.86	9.59	5.42	21.75	−4.868	<0.001
Toluene	317.76	211.41	122.50	353.93	89.93	31.58	12.61	141.32	−4.392	<0.001
Ethyl-benzene	40.91	21.00	14.05	53.33	12.09	3.92	1.91	16.68	−3.990	<0.001
m-xylene	113.85	58.44	35.85	140.56	28.44	7.11	3.64	42.87	−4.081	<0.001
p-xylene	37.18	21.92	13.85	42.46	10.62	3.06	1.55	16.72	−4.099	<0.001
o-xylene	57.51	29.18	18.16	76.79	15.67	4.14	2.37	23.98	−4.008	<0.001

BTEX: Benzene, toluene, ethyl-benzene, m-xylene, p-xylene and o-xylene. ***P*_25_**: 25th percentile. ***P*_75_**: 75th percentile.

**Table 3:** T-SOD, GSH, MDA, 8-OHdG, micronucleus rate, TM and OTM in refueling workers and office workers.

Oxidative Stress and Genetoxic Indicators	Refueling Workers (*n* = 200)	Office Workers (*n* = 52)
	Median (*P*_25_–*P*_75_)	Median (*P*_25_–*P*_75_)
T-SOD (U/mL)	42.97 (39.04–47.04)	48.70 (45.49–52.46)
GSH (mg/L)	6.91 (5.56–8.61)	8.85 (6.29–10.99)
MDA (μmol/L)	3.02 (2.55–3.70)	2.42 (2.09–2.77)
8-OHdG (mg/L) *	23.23 (15.87–39.21)	19.79 (13.54–25.58)
micronucleus rate (‰)	3.00 (1.00–8.00)	2.00 (0.25–3.00)
TM (μm)	0.094 (0.045–0.215)	0.064 (0.027–0.113)
OTM (μm)	0.160 (0.099–0.285)	0.104 (0.068–0.153)

* 8-OHdG was analyzed only in 57 refueling workers and 43 office workers. T-SOD: Total-superoxide dismutase. GSH: Glutathione. MDA: Malondialdehyde. 8-OHdG: 8-Hydroxydeoxyguanosine. TM: Tail moment. OTM: Olive tail moment.

**Table 4:** Multiple linear regression analysis of products of serum oxidative stress and genotoxicity end points with independent variables.

Dependent Variables	Independent Variables *	β (SE)	*t*	*p*
T-SOD	groups	−5.987 (0.972)	−6.159	<0.001
GSH	groups	−2.628 (0.551)	−4.768	<0.001
MDA	groups	0.778 (0.148)	5.240	<0.001
8-OHdG	groups	5.958 (2.606)	2.286	0.025
Micronucleus rate	groups	3.685 (0.683)	5.476	<0.001
	years of exposure	0.236 (0.021)	3.892	<0.001
TM	groups	0.103 (0.035)	2.897	0.004
OTM	groups	0.110 (0.033)	3.344	0.001

* Group assignment: Refueling workers for 1, office worker for 0. SE: Standard error.
